# International virtual confidential reviews of infection-related maternal deaths and near-miss in 11 low- and middle-income countries – case report series and suggested actions

**DOI:** 10.1186/s12884-022-04731-x

**Published:** 2022-05-23

**Authors:** Obiageli Okafor, Nathalie Roos, Abdulfetah Abdulkadir Abdosh, Olubukola Adesina, Zaynab Alaoui, William Arriaga Romero, Bouchra Assarag, Olufemi Aworinde, Luc de Bernis, Rigoberto Castro, Hassan Chrifi, Louise Tina Day, Rahel Demissew, María Guadalupe Flores Aceituno, Luis Gadama, Biruck Gashawbeza, Sourou Goufodji Keke, Philip Govule, George Gwako, Kapila Jayaratne, Evelyne Béwendin Komboigo, Bredy Lara, Mugove Gerald Madziyire, Matthews Mathai, Rachid Moulki, Iatimad Moutaouadia, Stephen Munjanja, Carlos Alberto Ochoa Fletes, Edgar Ivan Ortiz, Henri Gautier Ouedraogo, Zahida Qureshi, Zenaida Dy Recidoro, Hemantha Senanayake, Priya Soma-Pillay, Khaing Nwe Tin, Pascal Sedami, Dawit Worku, Mercedes Bonet, on behalf of the WHO Global Maternal Sepsis Study- Maternal Death and Near-miss Review Working Group, Sourou Goufodji Keke, Pascal Sedami, D. Vincent Batiene, Kadari Cisse, Evelyne Béwendin Komboigo, Henri Gautier Ouedraogo, Abdulfetah Abdulkadir Abdosh, Rahel Demissew, Biruck Gashawbeza, Ayalew Mariye, Thomas Mekuria, Filagot Tadesse, Fikremelekot Temesgen, Alula M. Teklu, Dawit Worku, Richard Adanu, Kwame Adu-Bonsaffoh, Philip Govule, Charles Noora Lwanga, Ama Asantewa Tamatey, William Enrique Arriaga Romero, María Guadalupe Flores Aceituno, Ligia María Palma Guerra, Carolina Bustillo, Rigoberto Castro, Carlos Alberto Ochoa Fletes, Bredy Lara, George Gwako, Alfred Osoti, Zahida Qureshi, Luis Gadama, Zaynab Alaoui, Bouchra Assarag, Hassan Chrifi, Rachid Moulki, Iatimad Moutaouadia, Hla Mya Thway Einda, Thae Maung, Myint Moh Soe, Khaing Nwe Tin, Olubukola Adesina, Chris Aimakhu, Olufemi Aworinde, Bukola Fawole, Zenaida Dy Recidoro, Hemali Jayakody, Kapila Jayaratne, Dhammica Rowel, Hemantha Senanayake, Mugove Madziyire, Thulani Magwali, Stephen Munjanja, Luc de Bernis, Louise Tina Day, Matthews Mathai, Edgar Ivan Ortiz, Priya Soma-Pillay, Mercedes Bonet, Obiageli Okafor, Nathalie Roos

**Affiliations:** 1 Harvard T. H Chan School of Public Health, Boston, MA USA; 2 Department of Medicine, Clinical Epidemiology Division, Karolinska Institute, Stockholm, Sweden; 3 Department of Obstetrics and Gynecology, St. Paul Hospital Millennium Medical College, Addis Ababa, Ethiopia; 4 Department of Obstetrics and Gynecology, University College Hospital, Ibadan, Nigeria; 5 Regional Directorate of Health, Settat region Casablanca, Morocco; 6 Department of the Gynecology and Obstetrics, Hospital Regional de Occidente, Quetzaltenango, Guatemala; 7 National School of Public Health, Rabat, Morocco; 8 Department of Obstetrics and Gynecology, Bowen University, Iwo, Nigeria; 9 Independent Consultant in International Maternal and Perinatal Health, 47300 Bias, France; 10 Hospital Doctor Roberto Suazo Cordova, La Paz, Honduras; 11 Department of Infectious Disease Epidemiology, Maternal and Newborn Health Group, London School of Hygiene & Tropical Medicine, London, UK; 12 Department of Obstetrics and Gynecology, Addis Ababa University, Addis Ababa, Ethiopia; 13 Obstetrics, Gynecology and Obstetrics Critical Care Unit, Hospital Regional de Occidente, Quetzaltenango, Guatemala; 14 Department of Obstetrics and Gynecology, College of Medicine, University of Malawi, Zomba, Malawi; 15 Center of Research in Human Reproduction and Demography, Cotonou, Benin; 16 Department of Epidemiology and Disease Control, School of Public Health, University of Ghana, Accra, Ghana; 17 Department of Obstetrics and Gynecology, University of Nairobi, Nairobi, Kenya; 18 Family Health Bureau, Ministry of Health, Colombo, Sri Lanka; 19 Training and Research Unit in Health Sciences, Nazi Boni University, Bobo-Dioulasso, Burkina Faso; 20 Ministry of Health of Honduras, Tegucigalpa, Honduras; 21 Clinical Trials Research Centre, University of Zimbabwe College of Health Sciences, Harare, Zimbabwe; 22 Independent Consultant in International Maternal and Perinatal Health, St John’s, Newfoundland and Labrador Canada; 23 Ben Msik Prefectural Hospital Center, Casablanca, Morocco; 24 Department of Obstetrics and Gynecology, University of Zimbabwe, Harare, Zimbabwe; 25 San Felipe Hospital, Tegucigalpa, Honduras; 26 Department of Obstetrics and Gynecology, University of Valle, Cali, Colombia; 27 National Center for Scientific and Technological Research: Ouagadougou, Center, Ouagadougou, Burkina Faso; 28 Department of Health, Disease Prevention and Control Bureau, Manila, Philippines; 29 Department of Obstetrics and Gynecology, Faculty of Medicine, University of Colombo, Colombo, Sri Lanka; 30 Department of Obstetrics and Gynecology, University of Pretoria and Steve Biko Academic Hospital, Pretoria, South Africa; 31 Department of Public Health, Maternal and Reproductive Health Division, Ministry of Health and Sports, Naypyitaw, Myanmar; 32 Ministry of Health, Cotonou, Benin; 33 Department of Sexual and Reproductive Health and Research, UNDP/UNFPA/UNICEF/WHO/World Bank Special Programme of Research, Development and Research Training in Human Reproduction (HRP), World Health Organization, 27 CH-1211 Geneva, Switzerland

**Keywords:** Audit, Infections, Maternal death, Near-miss, Perinatal, Virtual

## Abstract

**Background:**

Obstetric infections are the third most common cause of maternal mortality, with the largest burden in low and middle-income countries (LMICs). We analyzed causes of infection-related maternal deaths and near-miss identified contributing factors and generated suggested actions for quality of care improvement.

**Method:**

An international, virtual confidential enquiry was conducted for maternal deaths and near-miss cases that occurred in 15 health facilities in 11 LMICs reporting at least one death within the GLOSS study. Facility medical records and local review committee documents containing information on maternal characteristics, timing and chain of events, case management, outcomes, and facility characteristics were summarized into a case report for each woman and reviewed by an international external review committee. Modifiable factors were identified and suggested actions were organized using the three delays framework.

**Results:**

Thirteen infection-related maternal deaths and 19 near-miss cases were reviewed in 20 virtual meetings by an international external review committee. Of 151 modifiable factors identified during the review, delays in receiving care contributed to 71/85 modifiable factors in maternal deaths and 55/66 modifiable factors in near-miss cases. Delays in reaching a GLOSS facility contributed to 5/85 and 1/66 modifiable factors for maternal deaths and near-miss cases, respectively. Two modifiable factors in maternal deaths were related to delays in the decision to seek care compared to three modifiable factors in near-miss cases. Suboptimal use of antibiotics, missing microbiological culture and other laboratory results, incorrect working diagnosis, and infrequent monitoring during admission were the main contributors to care delays among both maternal deaths and near-miss cases. Local facility audits were conducted for 2/13 maternal deaths and 0/19 near-miss cases. Based on the review findings, the external review committee recommended actions to improve the prevention and management of maternal infections.

**Conclusion:**

Prompt recognition and treatment of the infection remain critical addressable gaps in the provision of high-quality care to prevent and manage infection-related severe maternal outcomes in LMICs. Poor uptake of maternal death and near-miss reviews suggests missed learning opportunities by facility teams. Virtual platforms offer a feasible solution to improve routine adoption of confidential maternal death and near-miss reviews locally.

**Supplementary Information:**

The online version contains supplementary material available at 10.1186/s12884-022-04731-x.

## Background

Obstetric infections are the third most common cause of maternal mortality, with the largest burden in low and middle-income countries (LMICs) (10·7%) compared with high-income countries (HICs) (4·7%) [1]. The true proportion of infection-related maternal deaths is most probably higher, as the estimates do not include deaths due to abortion-related or non-obstetric infections. The Global Maternal Sepsis Study (GLOSS), led by the World Health Organization (WHO), estimated that about 70 women per 1000 live births were admitted or already hospitalized with a maternal infection [2]. Among those, 11 women per 1000 live births had infection-related severe maternal outcomes (SMO: death or near-miss). The most common infections identified in the GLOSS study were urinary tract infections, endometritis, chorioamnionitis, abortion-related infections, and infections of the skin and soft tissue, in line with previous studies [3–5].

Infection-related maternal mortality and severe morbidity can have many contributing factors, including delays in the decision to seek care, arrival at the appropriate health facility, and provision of adequate and timely management [6]. Maternal and Perinatal Death Surveillance and Response (MPDSR), a process aimed at improving identification, reporting, and review of maternal deaths, is key for addressing health system issues and inadequacies to end preventable maternal mortality [6, 7]. Individual maternal death review is one of the cornerstones of MPDSR to assess the causes of death during pregnancy, around the time of childbirth, post-partum, or post-abortion, identify missed opportunities within the health system, and facilitate appropriate response, in a continuous surveillance cycle. However, it is crucial not only to avert future similar deaths but also to prevent acute severe complications and longer-term sequelae associated with experiencing a maternal near-miss (women who nearly died but survived a life-threatening complication that occurred during pregnancy, childbirth or 6 weeks postnatally [3]). Review of maternal near-miss cases may provide additional information on factors associated with occurrence of adverse outcomes, as they occur much more frequently than maternal deaths [8, 9].

Maternal deaths and near-miss reviews, when timely implemented with accurate and complete data [10], and in an environment of confidentiality, no-blame and professionalism, are useful in identifying contributing factors and formulating recommendations to improve services and quality of care [7, 11–13], and ultimately reduce all cause or cause-specific mortality and severe morbidity [14, 15], such as maternal infections [16, 17].

Considering the significance of infection-related maternal deaths and near-misses, and paucity of studies reporting on in-depth explorations of their causes and the surrounding circumstances in LMICs, this study sought to contribute to current knowledge on factors affecting the survival of pregnant or recently pregnant women after infection, using the GLOSS study [2].

This manuscript describes the results of an international, virtual confidential enquiry into maternal deaths and near-miss cases identified within the frame of the GLOSS study. The international external review committee analyzed causes of infection-related maternal deaths and near miss, identified clinical and non-clinical modifiable factors from before admission until death or discharge and generated suggested actions for facility level care improvement to avoid preventable infection-related maternal deaths and near-miss.

## Methods

### Study design and participants

**Fig. 1:**
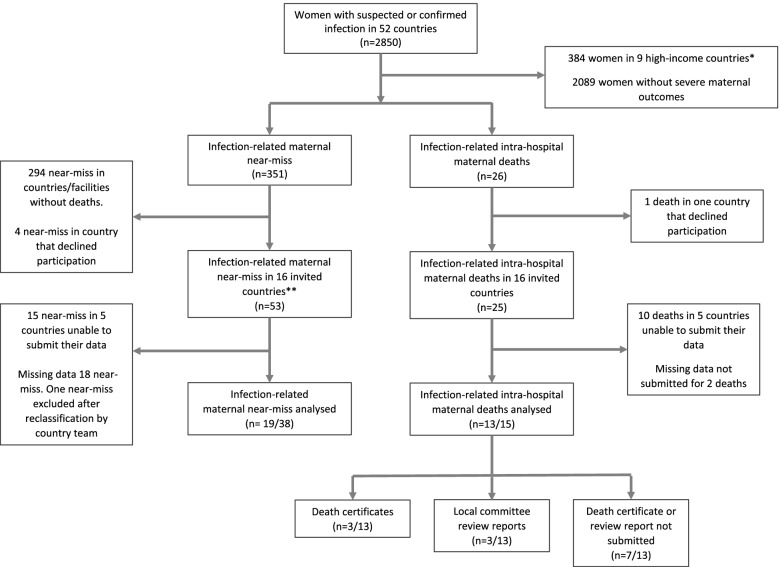
Study profile. *Note.* * No deaths reported. **Western European countries did not collect WHO near-miss criteria

The Global Maternal Sepsis Study (GLOSS) was a facility-based, prospective, inception cohort study implemented in health care facilities located in pre-specified geographical areas in 52 countries [18]. During a seven-day period (November 28 to December 04, 2017), all women admitted to or already hospitalized in participating facilities with suspected or confirmed infection during any stage of pregnancy through to 42 days after abortion or childbirth were included in the study. Participants were followed during their stay in the facilities until hospital discharge, transfer to another health facility not included in the study, or death. Among 2466 women recruited (Fig. 1) from 43 low- and middle-income countries (LMICs), a total of 26 infection-related maternal deaths and 351 infection-related near-miss cases were reported.

This is an extension of the GLOSS protocol, that includes additional data collection, and where all maternal deaths and near-miss cases from health facilities reporting at least one infection-related death were eligible for inclusion. A total of 16 countries, out of 17 eligible, expressed interest to participate in this study. Therefore, 25 infection-related maternal deaths and 53 near-miss cases from 25 health facilities were considered for inclusion. Cause of deaths were classified using the WHO ICD-MM (The WHO application of ICD-10 to deaths during pregnancy, childbirth, and puerperium: ICD-Maternal Mortality) system [19]. Near-miss cases were defined using the WHO criteria as a woman who nearly died but survived a life-threatening condition during pregnancy, childbirth, post-partum or post-abortion periods [3].

Ethical approvals were obtained from the WHO ethics review committee and as required by national or local entities.

### Data collection

Health facility information was available from the GLOSS study [18, 20]. As part of the preparation for facility inclusion for this study, we requested baseline data on facility characteristics related to local maternal death review processes and characteristics of the local committees shown in Additional file 1: Table S1, including existing systems to review deaths and near-miss, composition of the committee, organization of the meetings, documentation used to review or produced after the review process, findings dissemination channels and mechanism to follow up on the committee recommendations.

During the GLOSS study, information was collected from individual facility medical records on sociodemographic and obstetric characteristics of the woman; source of infections and management in the health facility; and maternal, perinatal, and neonatal outcomes. However, given that the design and initial objectives of the GLOSS study did not include a review component for maternal deaths and/or near-miss, comprehensive information for such reviews was not specifically collected. Additional data for this extended phase of the GLOSS study were requested using the form in Fig. S1, Additional file 1, from the facilities on: antenatal care received, mode of transport to the hospital, prior hospital admissions and referral including vital signs and laboratory measurement during hospital stay, timing of antimicrobials, additional diagnoses or management, managing multidisciplinary team composition, detailed information on cause of death and use of ICD-10 coding in case of death or near-miss. Data collection forms, the research protocol, and a manual of operation were translated from English into French and Spanish. Anonymized index case data collection forms were completed manually, scanned and transferred by the participating facility study coordinators, then entered into and managed using REDCap (Research Electronic Data Capture) electronic data capture tools [21, 22] by the WHO coordination team.

Index case anonymized available documents were collected from health facilities or local review committees, including anonymized copies of death certificates, autopsy reports, reports from local death reviews, or other documents relevant to each case.

### Virtual maternal and near-miss case review process

An external multi-country review committee was formed and tasked to perform independent assessments virtually of all included infection-related maternal deaths and near-miss cases. The external review committee consisted of 30 experts from 12 countries who indicated interest in taking part in the reviews, including GLOSS regional and country coordinators, and members of the country teams. All participants were health care providers, including gynecologists and obstetricians, internists and cardiologists, non-specialist medical doctors, nurses, and midwives. About half of the participants reported having extensive experience in review meetings, while half reported having little experience. Participants were divided into five multi-country review committees based on their preferred languages (English, French, and Spanish) and time zones. Five internationally recognized expert facilitators of maternal death and near-miss reviews and MPDSR were invited to serve as meeting moderators and were paired to the review committee groups. A team of WHO staff and consultants coordinated logistics for the review meetings.

Case summaries were prepared for each maternal death and near-miss case by the WHO coordination team using the template in Fig. S2, Additional file 1, that was adapted from the International Federation of Gynecology and Obstetrics (FIGO) Maternal Death Review clinical summary form [23]. Case summaries presented a main diagnosis, timing and chain of events, a narrative of the case management, outcomes, and facility characteristics. Distinct from the source clinical form, the case summary form used in this study contained questions that were specific to the management of an infection, for example, the date and time of the first antibiotic and the date and time of suspicion or diagnosis of infection. The case summary form also investigated the use of the International Classification of Disease (ICD) codes by facility review teams to classify cases of maternal deaths.

To assess gaps in management from before admission until death or discharge and document the review findings, a note-taking form in Fig. S3, Additional file 1, was adapted from Borchert et al. and Menéndez, et al. [24, 25]. The form provided an objective way to evaluate the severity of modifiable factors/gaps as minor, intermediate, or major, evaluate the severity of diagnosis discrepancies, and help review committee members think through the gaps as they read the cases before the meetings.

Over a three-month period, between August 31, 2020, and November 11, 2020, 20 virtual external review meetings were conducted. Each review meeting was scheduled for 75 minutes and facilitated by one of the international moderators. Maternal deaths and near-miss cases were grouped into three geographical groups based on the review languages and numbered sequentially. Using the numbers, the index cases were randomly allocated to committees outside the geographical area where the cases occurred. Two deaths and one near-miss cases were assigned for each meeting and the review committees were sent case summaries 10 to 14 days before the meeting. Individual members of the committees were tasked to prepare a presentation on one case to be presented at the virtual meetings and a facilitated discussion followed to build consensus. In cases where a team was unable to review all three cases assigned per session, the remaining case was reviewed in the subsequent meeting. Two assigned individuals from the coordination team took notes at the meetings to document the cause of death, missed opportunities and recommendations discussed.

### Analysis

We described the numbers and proportions of maternal demographic, obstetric and clinical characteristics, as well as complications, and outcomes for maternal deaths and near-miss cases.

The meeting notes for each case were reviewed by the coordination team and modifiable factors were identified and categorized following a structured process of thematic analysis [26], informed by themes defined in previously published work [24]. Factors that occurred prior to hospital admission, at admission, diagnosis, treatment, and discharge were included. New sub-themes were created where existing ones were not adequate. These included information on the circumstances that preceded maternal presentation to the GLOSS health facility such as antenatal care history and pre-existing comorbidities, the adequacy of the managing team at the GLOSS facility, and whether or not a facility review was conducted.

Modifiable factors identified during the external review meetings were then organized into the following categories using the three delays framework [27] - (1) deciding to seek appropriate medical help for an obstetric emergency; (2) reaching an appropriate facility; and (3) receiving adequate care when a facility is reached. Gaps that did not directly result from a delay to seek, reach, or receive adequate care were categorized under other clinical and non-clinical factors as per Table 2. Other clinical factors included antibiotics resistance and unexplained prolonged hospitalization. Non-clinical factors were related to the managing team (composition, mobilization, and collaboration), and local contextual factors and policies.

Recommendations were classified into five thematic areas following the three delays framework and consolidated across all reviews to avoid repetition. Recommendations addressing delays 1 and 2 were combined into a single thematic area due to the paucity of data for these delays. Recommendations related to delay 3 (receiving adequate care when a facility is reached) and other clinical factors were sub-divided into three thematic areas namely clinical and laboratory examination, diagnosis, and treatment. A fifth thematic area addressed the gaps related to the managing team.

Preliminary results and summary of the recommendations formulated during the review meetings were shared with the members of the five review committees in two joint virtual meetings providing an opportunity to discuss the relevance of findings and feasibility of recommendations across various local contexts.

## Results

There were 25 infection-related maternal deaths and 53 near-miss cases from 25 health facilities in 16 countries eligible for inclusion. However, only 11 countries submitted the necessary additional data on 13 out of 15 maternal deaths and 19 out of 47 near-misses (Fig. 1). Five country study teams were unable to submit their data due to factors including inability to trace the case folders, a demanding data collection process, staff changes that affected country study teams, and difficulty obtaining additional local ethical approvals. Three of the 11 countries submitted death certificates, and three submitted additional documentation. Only one country submitted both death certificates and additional documentation.

From the documented information available, most women with severe maternal outcomes (SMO) related to infection were between ages 20 and 35 (*n* = 7/13 maternal deaths and *n* = 14/19 near-miss) (Table 1). Two-thirds of SMOs occurred in the post-partum period (*n* = 6/13 deaths and *n* = 10/19 near-miss) and to multiparous women (*n* = 8/13 deaths and *n* = 12/19 near-miss). Close to a third of deaths (*n* = 4/13) and near-miss (*n* = 5/19) were from post-abortion complications. Among SMOs, at least one in every five had delivered by cesarean section with near-miss cases (*n* = 8/19) occurring almost twice as often in this group compared to maternal deaths (*n* = 3/13). Just over half of the women who died (*n* = 7/13) or had a near-miss (*n* = 10/19) were managed in intensive care (ICU) or in a high dependency unit (HDU). More than a third of women (*n* = 5/13) died within the first 48 hours of admission to the health facility. Two thirds of near-miss cases required more than a week of hospital admission (*n* = 13/19). Perinatal outcome was very poor – only 3/15 survived when there was a maternal death and 8/19 in the case of a near-miss. The results presented from the virtual review meetings are from 12 of the 13 maternal deaths as one death occurred within the first hour of presentation before the managing team commenced clinical management, and data was not sufficient to conduct a review of the case. Facility audits were conducted for only 2/13 maternal deaths and 0/19 near-miss cases (See Table S1, Additional file 1).

**Table 1:** Demographic, obstetric, clinical characteristics and outcomes of maternal deaths and near-miss cases

	Maternal deaths (***N*** = 13)	Maternal near-miss (***N*** = 19)
***Maternal age (years)***		
< 20	3	3
20–35	7	14
> 35	1	2
***Parity***		
Nulliparous	4	7
Multiparous	8	12
***Mode of end of pregnancy***		
Vaginal birth	2	3
Caesarean section	3	8
Abortion	4	5
Undelivered	2	3
***Period infection was identified***		
Antepartum	4	4
Intrapartum	1	3
Postpartum	5	7
Post-abortion	3	5
***Pregnancy status at death or near-miss (weeks)***		
Pregnancy	3	3
Postpartum	6	10
Post-abortion	4	6
***Perinatal outcome***^a^***(n = 10 in maternal deaths and n = 11 in near-miss cases)***		
Stillbirth	4	3
Intra-hospital early neonatal death^b^	2	0
Alive at end of follow up^c^	3	8
***Maternal admission to intensive or high dependency care***		
Yes	7	10
No	6	9
***Time from admission to maternal death or discharge***		
≤ 48 hours	5	0
2–7 days	6	6
1–6 weeks	1	13

^a^Includes only births. Includes twins

^b^*n* = 2 of 5 live births

^c^Live newborn discharged from hospital or day 7 after birth if mother is still hospitalized

The external review committee identified 151 modifiable factors from the available information, among which 85 were from death reviews and 66 were from near-miss reviews (Table 2) - the most common modifiable factors were related to delays in receiving adequate care in the facility. Between three and 12 factors were identified per maternal death, and between zero and seven factors per near-miss (See Table S2, Additional file 1).

**Table 2:** Documented clinical and non-clinical modifiable factors identified in 12 maternal deaths and 19 near-miss cases

Modifiable factors		
	**Maternal deaths (*N*** **= 85)**	**Near-miss** **(*N*** **= 66)**
	**n (%)**	**n (%)**
Delay in decision to seek care	**2 (2.4)**	**3 (4.5)**
Late antenatal care	0	2
Delay in deciding to seek appropriate medical help	2	1
Delay in reaching the GLOSS facility^a^	**5 (5.9)**	**1 (1.5)**
Delay in referral to a higher-level facility	5	1
Delay in receiving care	**71 (83.4)**	**55 (83.3)**
*Prior to arrival to the level 3 facility*	*6 (7.0)*	*8 (12.1)*
Delivered by a traditional birth attendant	1	3
Inadequate management of preexisting conditions	2	3
Missing referral information	3	2
*Clinical and laboratory examinations*	*20 (23.5)*	*17 (25.8)*
Inadequate clinical examination at admission	3	2
Inadequate monitoring after admission	5	2
Missing or delayed microbiological culture^b^	7	7
Missing or delayed other laboratory & diagnostics	5	6
*Diagnosis*	*13 (15.3)*	*12 (18.2)*
Incorrect working diagnosis during case management	6	2
Incomplete main diagnosis	3	0
Delayed diagnosis	0	1
Criteria for diagnosis not met	4	4
Source of infection not identified	0	2
Near-miss criteria (based on WHO definition) not met^c^	0	3
Missing diagnosis	0	0
*Management*	*32 (37.6)*	*18 (27.3)*
Incomplete clinical management of infection^d^	2	1
Suboptimal use of antibiotics^e^	9	6
Delay or overuse of other medications^f^	5	6
Insufficient intravenous fluid	2	0
Insufficient blood transfusion	2	0
Delayed ICU/HDU admission^g^	6	3
Delayed control of the source of infection	3	1
Missing or delayed interventions^h^	3	1
Other clinical factors	**1 (1.2)**	**4 (6.1)**
Antimicrobial resistance	1	1
Unexplained prolonged hospitalization^i^	0	3
Non-clinical factors	**6 (7.1)**	**3 (4.5)**
Incomplete multidisciplinary team^j^	3	2
Delayed mobilization of the managing team	1	0
Restrictive abortion policies and legislation	1	0
Stigma of preexisting condition as a potential care barrier	0	1
Discharge against medical advice	1	

^a^All GLOSS facilities in this study are level 3 health facilities

^b^Bacterial culture from blood, urine, respiratory tract

^c^Near-miss were identified by countries according to the GLOSS protocol criteria. However, cross-check by the review team revealed 3 cases that did not meet the criteria

^d^Management focused on other conditions and not the infection

^e^Includes delayed, wrong and overuse of antibiotics

^f^Antivirals, antifungal, steroids, antimalaria, diuretic and inotropic agents

^g^ICU=Intensive Care Unit; HDU = High Dependency Unit

^h^Interventions include induction/birth, manual removal of placenta, dilation and curettage, emergency laparotomy, hysterectomy

^i^Prolonged hospitalization was assessed based on the reported improvement in the clinical status of the woman

^j^Multidisciplinary team characterized by a minimum of an obstetrician-gynecologist and anesthesiologist plus any additional specialist needed depending on the case

Key: GLOSS-Global Sepsis Study

**Fig. 2:**
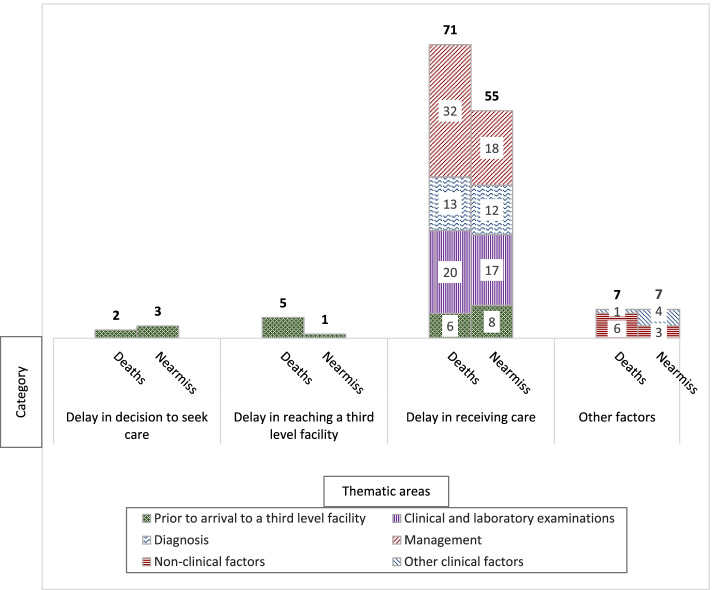
Number of modifiable factors identified in the review of 12 maternal deaths and 19 near-miss. *Note.* Bold numbers are total. Other clinical factors include antimicrobial resistance and unexplained prolonged hospitalization. Non-clinical factors include incomplete multidisciplinary team, delayed mobilization of the managing team, restrictive abortion policies and legislation, stigma of preexisting condition as a potential care barrier, discharge against medical advice

The delay in receiving care (delay 3) was considered by the committee to have contributed to 71 of the 85 modifiable factors for maternal deaths (Fig. 2), and 55 of the 66 modifiable factors for near-miss cases. Among maternal deaths, five of the 85 modifiable factors were associated with a delay in reaching a GLOSS facility in a timely manner (delay 2) compared to one of the 66 factors in near-miss cases. A delay in the decision to seek care (delay 1) was documented in two of the 85 modifiable factors identified for maternal deaths compared to three of the 66 modifiable factors in near-miss cases. Other clinical and non-clinical factors contributed each to seven of 85 modifiable factors for maternal deaths and 66 near-miss cases.

Inadequate or delayed treatment made up 32 of 71 modifiable factors related to a delay in receiving care (delay 3) for maternal deaths compared to 18 of 55 factors for near-miss cases (Fig. 2). Clinical and laboratory examination issues contributed to 20 of 71 modifiable factors in maternal deaths and 17 of 55 factors in near-miss cases. Diagnosis-related problems were higher in near-miss cases at 12 of 55 modifiable factors than maternal deaths with 13 of 71 factors. Table 2 details the modifiable factors identified during the review.

Table S3, Additional file 1 compares the causes of death reported in the facility records with re-assigned causes of death after review by the GLOSS coordination team. A cause of death was not recorded in 2 of the 13 deaths reviewed. For 4 of the 11 deaths, the review committee agreed with the reported underlying cause of death as an infection but provided a more detailed cause of death. At the facility level, only 4 of the 13 deaths were assigned a cause of death using the ICD coding system, 2 using ICD-10 and 2 using ICD-MM.

Based on the review findings, the external review committee generated a broad set of recommendations for improved prevention and management of maternal infections and sepsis presented in Table 3.

**Table 3:** Suggested actions for improved prevention and management of maternal infections and sepsis identified from reviews

Thematic area	Modifiable factors	Suggested actions
**1. Prior to arrival to a level 3 facility**	***1.1. Need for improved pre-conception care***	• Improve family planning counselling and contraceptive services during antenatal and postpartum care to promote birth spacing and planning of pregnancy, especially among high-risk women
	***1.2 Need for improved antenatal care***	• Use of risk criteria and nationally adapted guidance to ensure high quality, timely and complete antenatal care contacts for early identification of high-risk pregnancies
		• Prioritize detection, and prompt treatment and monitoring of common infections during antenatal care
	***1.3. Access to high quality abortion and post-abortion care***	• Ensure access to safe abortion and post-abortion care services under the supervision of trained providers
	***1.4 Delay in seeking medical care***	• Create partnerships with traditional birth attendants (TBAs) to define and agree on their roles in supporting and promoting safe health practices during pregnancy and childbirth, including early referral and access to safe abortion and post-abortion services
		• Promote facility-based childbirth during antenatal care with childbirth preparedness counselling and ensure physical, financial, and culturally appropriate access to skilled and high-quality facility-based care
		• Develop and implement a behavior change communication plan for women and their communities with regards to responding to danger signs during pregnancy, postpartum and post-abortion to ensure timely facility-based consultation and care by a trained health care provider
	***1.5 Delay in referral to higher level of care within or outside the facility***	• Ensure early recognition of the need for higher level of care for pregnant or recently pregnant women at the time of admission or during their stay in a health facility to allow timely and safe referrals
		• Improve communication between health care facilities, prior to, during and after referrals, including feedback to the referring facility (both positive and negative) on the referral processes and health outcomes
		• Include referring health facilities and health providers in the maternal death and near-miss reviews to share insights into relevant medical history or delays that occurred prior to admission for mutual learning
**2. Clinical and laboratory examinations**	***2.1. Need for improved clinical examination at admission and monitoring***	• Introduce clinical early warning scoring systems at admission for assessment of maternal infection severity and sepsis
		• Triage all pregnant and postpartum/post-abortion women at admission to ensure the right level of care for critically ill women
		• Ensure routine regular and complete monitoring of vital signs at admission with regular follow-up
	***2.2. Microbiological culture taking***	• Obtain blood culture samples, and samples from other suspected infection foci, prior to antibiotic treatment, in all cases of suspected maternal sepsis
	***2.3. Missing or delayed laboratory or other diagnostics***	• Use adequate and complete laboratory tests to support clinical diagnosis, adequate management, and monitoring of the woman’s health condition
		• Use available imaging (e.g., X-ray, ultrasound) to complement clinical diagnosis and support adequate management
**3.Diagnosis**	***3.1. Correct diagnosis***	• At arrival at the higher levels of care, re-evaluate initial diagnosis from the referring facility to influence management and outcomes
		• Improve identification of infection source by ensuring a comprehensive clinical history, examination, laboratory investigation and imaging
**4. Treatment and management**	***4.1. Antibiotic resistance and stewardship***	• Ensure use of adequate antibiotic class and dose tailored to the source and severity of infection, including use of broad-spectrum antibiotics only when necessary
		• Review antibiotic management based on results from bacterial culture, antimicrobial resistance profile, and clinical presentation, including avoiding changes in prescription without clear microbiological or clinical indication
		• Document and monitor reasons for changes in antibiotics prescriptions, including for example availability of microbiological results, changes in clinical status, availability of antibiotics
	***4.2. Delayed interventions***	• Remove or treat the identified infection foci as rapidly as possible
		• Administer antibiotics without delay to critically ill women after securing adequate culture samples
		• In critically ill septic pregnant or recently pregnant women, ensure intravenous fluid resuscitation is commenced immediately on arrival to the hospital
		• Build capacity of health care providers for performance of timely and safe cesarean sections and management of post-surgical complications
	***4.3. Documentation and follow up***	• Ensure documented medical history in early pregnancy, including pre-existing conditions and risk factors for assigning the adequate level of care
		• Complete routine inpatient documentation in medical records for clinical history, clinical findings, laboratory results, treatments (dose, timing), timing of interventions, care management steps and other investigations
		• Link woman-baby medical records and include maternal and newborn health outcomes as part of woman-baby dyad centered care, simultaneously where possible.
		• Provide comprehensive discharge education to help women and families recognize danger signs after birth and particularly after cesarean section, for wound care and where to seek care if complications arise
**5.Managing team**	***5.1. Unclear multidisciplinary care guidelines***	• Build capacity of health care providers including at primary health care level in the recognition of danger signs of critically ill women, rapid management, and monitoring for infection-related complications.
		• Establish clear criteria for when multi-disciplinary teams should manage pregnant or recently delivered women with infection in tertiary level hospitals

## Discussion

This global virtual review, of 13 infection-related maternal deaths and 19 near-miss cases from 11 LMICs, highlights important gaps in quality standards of care related to delays in establishing diagnosis and appropriate management, including clinical and laboratory examinations. Information on care prior to presentation at the health facilities was often lacking or incomplete. Missed opportunities were exacerbated by antenatal care interventions not fully implemented, delayed referrals to higher level facilities, incomplete multidisciplinary teams as well as gaps in conducting reviews and disseminating findings to health facilities and staff involved in care.

Delays in the recognition of early warning signs of clinical deterioration, diagnosis, and prompt management of maternal infection and sepsis were common findings for both deaths and near-miss cases reviewed, in line with findings from previous infection-related deaths reviews in settings with limited resources [16, 28]. Utilization of clinical early warning systems globally, characterized by abnormal clinical observations of consciousness level, temperature, pulse rate, respiratory rate and blood pressure, have been shown to be an important tool for prompt diagnosis [29], better monitoring, and improved management of pregnant or recently pregnant women admitted with infection [30]. The external review committee recommended the use of early warning signs systems as part of the essential quality monitoring to evaluate the severity of maternal infection and assess the need for higher level care at admission or during hospital stay.

Gaps in diagnosis and management of maternal infections and sepsis could have resulted from delayed or absent microbiological cultures and relevant laboratory tests and imaging. Notably, among maternal deaths and near-miss cases with delayed or absent culture results, the sources of infection were likely incorrectly identified in two deaths and three near-miss cases and unidentified in one death. Similarly, others have reported lack of cultures in up to 25% of sepsis-related maternal deaths [16, 28]. While the reasons behind absent microbiology cultures were not documented in our study, others cite rapid deterioration of the woman’s clinical condition, lost samples, missing results from medical records, and out of pocket costs [16], as some of the reasons. While reiterating the importance of collecting blood culture samples prior to antibiotic treatment in all suspected maternal sepsis cases, the external review committee recommended that imaging and other relevant laboratory tests are also essential to enable diagnosis and support adequate monitoring and management.

Suboptimal use of antibiotics was a significant contributor to gaps in treatment identified for most of the maternal deaths and near-miss cases. Early administration of appropriate antimicrobial agents provide the most benefit in sepsis when accompanied by high quality supportive care – e.g. fluid resuscitation [30]. However, antimicrobial use with unsupported frequent and erratic changes without microbiological or clinical indication and removal of the infection source can be detrimental and contribute to the emergence of antimicrobial resistance [31]. While the external review committee agreed that ideally an antibiotic sensitivity profile ought to guide the choice of and switch between antimicrobial agents, they also recognized situations where the administration is justified when such a sensitivity profile is not possible or poses substantial delay in cases of severe infections or sudden deterioration in the clinical presentation. In those instances, adequate documentation of the reasons in medical records was encouraged. Members of the external review committee who had noted similar findings related to provision of quality care in their local maternal death audits highlighted a potential connection with health system gaps. For instance, a correlation between misdiagnosis and the absence or limitations of good laboratory facilities such as in primary level birthing centers; or delay in administration of antibiotics linked to a shortage of antimicrobial agents in the hospital pharmacy.

Significantly increased risk of fetal and neonatal deaths have been reported in countries where systemic infections/ sepsis are among the leading causes of maternal morbidity and mortality [32, 33]. Extremely low survival rates of the babies among the reviewed cases of maternal deaths (3/15) and maternal near-miss (8/19) support the association between SMO and poor perinatal outcomes [34]. Beyond coverage of essential interventions, delays in quality care implementation and lack of comprehensive supportive care are hypothesized contributors to maternal outcomes, and in turn, perinatal survival [32]. Linking maternal and fetal records with maternal and perinatal death surveillance and response can strengthen the provision of comprehensive supportive care to the mother-baby dyad.

We were also able to identify issues in the process of conducting reviews. At the facility level, the cases we reviewed demonstrated that there was no documented evidence that the majority of deaths/ near-miss cases had been reviewed, despite an established maternal death review processes reported in two-thirds of the included facilities. A majority of countries have policies in place for maternal death notification and review, yet a gap remains when examining the steps beyond this, including reviewing and reporting at an aggregate level, disseminating findings and recommendations, and involving civil society and communities [35]. While an understanding of the MPDSR process is fundamental to conducting reviews, the external review committee stressed that experience is not needed to get started. As reflected by the range of experience among members, the committee underscored that capacity building occurs through an iterative process of learning that occurs as teams participate in and incorporate the review meetings into the clinical management of cases and self-evaluation. The committee also stressed that a no-blame culture is critical to the successful implementation of reviews and response [7, 36], and noted that the inclusion of a near-miss case among the regular death reviews boosted the morale of the team.

Among the reviewed cases, the committee recognized a need to involve the referral facilities and staff in the review and dissemination process. Through the virtual platform, reviewers would be able to participate from any location, mitigating distance and manpower constraints which are barriers to maternal deaths audits [37].

Recommendations from our study are particularly relevant given the shortage of written protocols specific to the prevention or management of infection related SMO in LMIC contexts. Consistent with existing standard sepsis guidelines [30], the proposed recommendations reiterate the main issues continue to be prompt identification, monitoring, initial resuscitation with intravenous fluids, diagnosis using appropriate routine microbiologic cultures, early treatment with appropriate antimicrobials and rapid control of the source of infection. Close collaboration with other medical disciplines [16] especially in cases of non-obstetric causes of sepsis, competent and motivated staff [10] as well as a more thorough implementation of the review process by managing and referral facilities and staff are also important to optimize maternal outcomes.

### Strengths and limitations

To our knowledge, this is the first multi-country virtual maternal deaths and near-miss review case series. Due to the multinational composition of the external review committees and random assignments of anonymized cases to each committee, the committee members rarely encountered cases from their countries. Consequently, the nature of the review meetings enabled compliance with the basic principles of good practice of clinical audits including the confidentiality of information and the principles of no name, no shame, and no blame [8, 38]. Multi-country participation led to very rich discussions on how management differs between contexts and strong cross-country learning. One of such new learning for some was a minute of silence observed at each review meeting to humanize and honor the memories of the women that died. Interactions with members of other country teams and the experience of internationally recognized moderators enabled capacity strengthening.

Although the cases had originally been identified for the GLOSS research study, medical records information on care at the GLOSS facilities and prior to presentation were often lacking or incomplete and may explain why the majority of our recommendations focus on delay in receiving care. Efforts to obtain additional information on the missing data from other sources were unsuccessful. Incomplete or inadequate routine patient case notes documentation affects not only this study but is the reality in many settings, impeding clinical decision making and opportunities for audit and quality improvement efforts. Our study demonstrated the urgency to invest so that “Every woman and newborn has a complete, accurate, standardized medical record during labour, childbirth and the early postnatal period” ([10], p.41). Similar challenges with poor record keeping and lost records are reported in other obstetric audits [16, 37, 38]. Records for two maternal deaths and 18 near-miss cases were not found in the facility and there was a lack of consensus on the circumstances surrounding one maternal death. Such shortcomings in record keeping may undermine the quality of the information and review conclusions [39], as cases were analyzed and classified by the expert committee based on available information from medical records.

One of the study objectives was to compare findings and recommendations of local committee reviews and those from an external review committee. An extensive comparison was impossible given the low rates of internal audits conducted at the facilities.

Lastly, external reviewers may not have been completely familiar with the context and the facility managing teams were not available for clarifications during the review which could have affected interpretation of events by the reviewers. In addition, the confidential nature of the reviews limited the ability of the reviewers to probe further into the local context, therefore the ensuing recommendations were not specific to each death or near-miss or to the facility or country involved.

## Conclusion

The lack of precision in management due to limited continuous clinical assessment, use of clinical skills and laboratory examinations contribute to missed opportunities in the provision of high-quality care to prevent and manage infection related SMO, and shortfalls in maternal death and near-miss review processes in facilities in LMICs. Poor uptake of maternal deaths and near-miss reviews suggests missed learning opportunities by facility teams. Routine confidential enquiries and review meetings should be incentivized at the facility level as part of quality improvement efforts. This study confirms the feasibility of multinational maternal death and near-miss reviews using a virtual platform. While locally conducted reviews would allow for more detailed and context specific recommendations, virtual review meetings allow for a confidential approach to reviewing deaths across a diverse number of settings. The authors recognize the need for additional evaluations to assess the quality of the review process, compare and validate findings, assess the feasibility of implementing and adapting actionable recommendations to different contexts.

## Supplementary Information

**Additional file 1.** Supplementary appendix to “International virtual confidential reviews of infection-related maternal deaths and near-miss in 11 low- and middle-income countries – Case report series and suggested actions. Study forms used for data collection and during the external review meetings. Results of a baseline assessment of maternal death review (MDR) committees and practices in participating GLOSS facilities. Additional information on the causes of deaths and distribution of modifiable factors described in this study.

## Data Availability

The data used for this analysis can be made available upon reasonable request, in accordance with the GLOSS research group data sharing policy and WHO policy of data use and data sharing. For further information, contact the corresponding author.

## Abbreviations

LMICS: Low-and Middle-Income Countries
HICs: High Income Countries
GLOSS: The Global Maternal Sepsis Study
WHO: World Health Organization
SMO: Severe Maternal Outcomes
MPDSR: Maternal and Perinatal Death Surveillance and Response
WHO ICD-MM: The WHO application of ICD-10 to deaths during pregnancy, childbirth, and puerperium: ICD-Maternal Mortality
REDCap: Research Electronic Data Capture
FIGO: The International Federation of Gynecology and Obstetrics
ICD: International Classification of Disease
ICU: Intensive care
HDU: High dependency unit
