# Supplementary material for: International virtual confidential reviews of infection-related maternal deaths and near-miss in 11 low- and middle-income countries – case report series and suggested actions
Source: BMC Pregnancy Childbirth. 2022 May 23;22:431. doi: 10.1186/s12884-022-04731-x (PMC9128080; doi:10.1186/s12884-022-04731-x)
Supplement: Supplementary file 1 — Additional file 1. Supplementary appendix to “International virtual confidential reviews of infection-related maternal deaths and near-miss in 11 low- and middle-income countries – Case report series and suggested actions. Study forms used for data collection and during the external review meetings. Results of a baseline assessment of maternal death review (MDR) committees and practices in participating GLOSS facilities. Additional information on the causes of deaths and distribution of modifiable factors described in this study. [file 12884_2022_4731_MOESM1_ESM.pdf]

Supplementary appendix

International virtual confidential reviews of infection-related maternal deaths and near-miss in 11 low- and middle-income countries – Case report series and suggested actions

Supplementary Material

Figure S1: GLOSS maternal death and near-miss data collection form

World Health Organization

GLOBAL MATERNAL SEPSIS STUDY - DEATHS AND NEARMISS

INDIVIDUAL FORM

ALL MATERNAL DEATHS AND NEARMISS WITH INFECTION INCLUDED IN THE STUDY

Section A & B Care before/during admission (March 2019 - page 1/8)

Global Maternal Sepsis Study

Please use a black or blue ball-point pen. Please refer to instructions at the end of the form

Country code ☐☐☐ **Hospital ID** ☐☐ **Participant number** ☐☐

A) CARE BEFORE ENTERING THE STUDY

Unknown / No / Yes

1) Did the woman receive antenatal care? ☐ ☐ ☐

1.1) If yes, total number of antenatal visits ☐☐ *(unknown=99)*

1.2) Date of first antenatal visit d d m m y y *(unknown=99/99/99)*

2) Is there a history of any of the following in the previous 14 days before entering the study?
(previous to date of Q15 in GLOSS form - October 2017)

	Unknown	No	Yes
a) Abdominal pain (excludes contractions)	☐	☐	☐
b) Abnormal vaginal discharge	☐	☐	☐
c) Sore throat/cough	☐	☐	☐
d) Chest pain	☐	☐	☐
e) Dysuria	☐	☐	☐
f) Vomiting/diarrhoea	☐	☐	☐
g) Flu-like symptoms	☐	☐	☐
h) Mastitis	☐	☐	☐
i) Caesarean section wound infection	☐	☐	☐
j) Urinary Tract Infection	☐	☐	☐
k) Malaria	☐	☐	☐
l) Other infection	☐	☐	☐

l.1) If other, specify:

____

____

If No to all above, go to Q3

2.1) If Yes, did she receive treatment for the condition(s) in the previous 14 days before entering the study?

Unknown ☐ No ☐ Yes ☐

2.2) If yes, any prescription of antimicrobials in the previous 14 days before entering the study?:

	Unknown	No	Yes
a) Antibiotics	☐	☐	☐
b) Antifungals	☐	☐	☐
c) Antivirals	☐	☐	☐
d) Antimalarials	☐	☐	☐

If Yes, specify:

____

____

3) Any hospitalisation in the previous 14 days before entering the study? Unknown ☐ No ☐ Yes ☐
(excluding for childbirth)

3.1) If Yes, date of last admission d d m m y y Date of discharge d d m m y y
(unknown=99/99/99)

4) Main reasons of last hospitalisation in the previous 14 days before entering the study *(excluding for childbirth)*

____

____

B) ENTRY IN THE STUDY

5) Date and time of arrival at facility
(or admission if arrival not recorded)
(date of Q1 in GLOSS form - October 2017)

d d m m y y h h m m
(unknown=99/99/99) *(00:00-23:59 hrs)*

6) Did the woman receive care before arriving in the facility?

	Unknown	No	Yes
a) At home / community	☐	☐	☐
b) In ambulance	☐	☐	☐
c) In other facity before referral	☐	☐	☐

7) Was she referred more than once before arriving to this facility? Unknown ☐ No ☐ Yes ☐ .
If No or unknown go to Q10

8) Date and time of last transport before arrival at this facility
d d m m y y h h m m
(unknown=99/99/99) *(00:00-23:59 hrs)*

9) What was the mode of transportation used in last referral before arriving to this facility? *(tick one)*

- a) Public transport ☐
- b) Family transport ☐
- c) Non-medicalised formal transport ☐
- d) Ambulance with the facility ☐
- e) Ambulance called from other facility ☐
- f) Other ambulance service ☐
- g) A private or commercial transport vehicle ☐
- h) Unknown ☐

10) Diagnosis at admission to this facility

	No	Yes
a) Placentation abnormality *(praevia/accreta/increta/percreta/abruption)*	[ ]	[ ]
b) Antenatal haemorrhage	[ ]	[ ]
c) Pre-eclampsia	[ ]	[ ]
d) Eclampsia/HELLP syndrome	[ ]	[ ]
e) Gestational diabetes	[ ]	[ ]
f) Preterm labour	[ ]	[ ]
g) Prelabour rupture of membranes	[ ]	[ ]
h) Abortion/miscarriage	[ ]	[ ]
i) Pospartum/postabortion haemorraghe	[ ]	[ ]
j) Infection	[ ]	[ ]
k) Labour	[ ]	[ ]
l) Chronic disease	[ ]	[ ]
m) External injury in pregnancy	[ ]	[ ]
n) Unknown diagnosis	[ ]	[ ]
m) Other pregnancy complications	[ ]	[ ]

m.1) If other, specify:

____

____

C) CARE AFTER SUSPICION/DIAGNOSIS OF INFECTION

11) Date and time of infection suspicion /diagnosis

(date of Q15 in GLOSS form - October 2017)

d d m m y y | h h m m

(unknown=99/99/99) *(00:00-23:59 hrs)*

12) At suscipion/diagnosis of infection, which of the following vital signs were measured?

	No	Yes
a) Heart rate	[ ]	[ ]
b) Respiratory rate	[ ]	[ ]
c) Blood pressure	[ ]	[ ]
d) Temperature	[ ]	[ ]
e) Mental status	[ ]	[ ]

13) How often were vital signs recorded in the following 24h after suspicion/diagnosis of infection ? *(tick one)*

- a) < 1x/24h [ ]
- b) 1-2x/24h [ ]
- c) every 8h [ ]
- d) at least every 6h [ ]

14) Professionals involved in the management of the woman in first 24h after suspicion/diagnosis of infection ? *(tick all that apply)*

- a) Midwife [ ]
- b) Obstetrics specialist [ ]
- c) Obstetrician in training (resident) [ ]
- d) Internal Medicine specialist [ ]
- e) Physician who can perform caesarean section [ ]
- f) Critical care specialist [ ]
- g) Anaesthesiologist [ ]
- h) Anaesthetist (nurse/ paramedics) [ ]
- i) Infectious disease specialist [ ]
- j) Paediatrician [ ]

15) Was a decision taken to relocate the woman in the after suspicion/diagnosis of infection ?

	Not available	No	Yes
a) Admit to ICU	[ ]	[ ]	[ ]

a.1) If yes, date and time the decision was taken for the first time:

d d m m y y | h h m m

(unknown=99/99/99) *(00:00-23:59 hrs)*

	Not available	No	Yes
b) Admit to high dependency care unit/bed	[ ]	[ ]	[ ]

b.1) If yes, date and time the decision was taken for the first time:

d d m m y y | h h m m

	Not available	No	Yes
c) Theatre	[ ]	[ ]	[ ]

c.1) If yes, date and time the decision was taken for the first time:

d d m m y y | h h m m

	Not available	No	Yes
d) Transfer to higher level facility	[ ]	[ ]	[ ]

d.1) If yes, date and time the decision was taken for the first time:

d d m m y y | h h m m

16) List first three antimicrobials received for treatment of infection, date and start time *(refer to list)*

(Refer to Q24 in GLOSS form - October 2017)

16.1) ____________________

start date d d m m y y | start time h h m m

(unknown=99/99/99) *(00:00-23:59 hrs)*

16.2) ____________________

start date d d m m y y | start time h h m m

16.3) ____________________

start date d d m m y y | start time h h m m

D) POSTPARTUM/POSTABORTION HAEMORRHAGE

17) Did the woman have postpartum /postabortion haemorrhage? **No** [ ] **Yes** [ ]

(Refer to Q26 in GLOSS form - October 2017)

If No, go to Q24

17.1) If yes, Date and time of first symptoms/diagnosis

d	d	m	m	y	y		h	h	m	m

18) Where was the diagnosis made?

	No	Yes
a) Community/home		
b) Emergency room		
c) Labour/abortion ward		
d) Postnatal/postabortion ward		
e) Theatre		
f) Intensive care /high dependecy unit		
g) Other healthcare facility		
h) During transfer to this facility		
i) Other, specify:		

____

19) Was blood lost measured/ estimated? No [ ] Yes [ ]

If No, go to Q21

19.1) If yes, How much blood was lost within 24 hours? [ ][ ][ ][ ] *ml*

20) How was blood loss estimated?

	No	Yes
a) Visual estimation		
b) Direct collection and measurement		
c) Direct collection and weighing		
d) Blood sampling		
e) Other		

21) What was the cause of the haemorrhage?

	No	Yes
a) Uterine atony		
b) Vaginal/perineal/cervical tear		
c) Retained placenta/product of conception		
d) Uterine infection		
e) Uterine rupture		
f) Extension of CS incision		
g) Trauma		
h) Coagulopathy		
i) Unknown		
j) Other		

22) Did the woman receive medical treatment for postpartum/postabortion haemorrhage? **No** [ ] **Yes** [ ]

(Refer to Q28a in GLOSS form - October 2017)

If No, go to Q25. If Yes, specify:

a) Oxytocin No [ ] Yes [ ]

a.1) Total dose (UI) [ ][ ][ ]

a.2) Route IM [ ] IV [ ] Unknown [ ]

b) Ergometrine No [ ] Yes [ ]

b.1) Total dose (mcg) [ ][ ][ ][ ]

b.2) Route IM [ ] IV [ ] PO [ ] Unknown [ ]

c) Misoprostol No [ ] Yes [ ]

c.1) Total dose (mcg) [ ][ ][ ][ ]

c.2) Route IM [ ] IV [ ] PO [ ] Unknown [ ]

23) Did the woman receive other treatments for postpartum/postabortion haemorrhage? **No** [ ] **Yes** [ ]

(Refer to Q28b - j in GLOSS form - October 2017)

If No, go to Q24

If Yes, provide date and time

	d	d	mo	mo	h	h	mi	mi
a) Balloon/condom tamponade								
b) Uterine packing								
c) Uterine compression sutures								
d) Uterine artery embolization								
e) Uterine artery ligation								
f) Laparotomy								
g) Blood products / transfusion								
h) Central venous catheter								
i) In-dwelling urinary catheter								
j) Uterine massage								
k) Manual revision of the uterus								
l) Manual removal of the placenta								
m) Fluids								

(00:00-23:59 hrs)

E) PRE-ECLAMPSIA, ECLAMPSIA, HELLP

24) Was she a known hypertensive? Unknown [ ] No [ ] Yes [ ]

(including pregnancy-related hpertension)

24.1) If yes, was the woman receiving any treatment? Unknown [ ] No [ ] Yes [ ]

24.2) If yes, specify treatment:

____

24.3) If yes, was blood pressure controlled under treatment? Unknown [ ] No [ ] Yes [ ]

25) Did the woman have an acute episode of hypertension on admission/during this hospital stay? No [ ] Yes [ ]

25.1) If yes, date and time acute episode started

d	d	m	m	y	y		h	h	m	m

(unknown=99/99/99) *(00:00-23:59 hrs)*

25.2) Highest blood pressure during acute episode

SBP [| |] mmHg DBP [| |] mmHg

26) Did the woman have any hypertension-related complications? No [ ] Yes [ ]

If No, go to 28

26.1) If Yes, provide date and time of first presentation

	d	d	mo	mo	h	h	mi	mi
a) Proteinuria								
b) Epigastric pain								
c) Headache								
d) Oedema								
e) Oliguria								
f) Convulsions								
g) Dyspnea								
h) Cyanosis								
i) Pulmonary oedema								
j) Visual symptoms								
k) Altered reflexes								

27) Did the woman receive any interventions to manage hypertension? If No, go to 32 No [ ] Yes [ ]

27.1) If Yes, provide date and time of initiation

	d	d	mo	mo	h	h	mi	mi
a) Antihypertensives								
b) Magnesium sulphate								
c) Anticonvulsivants								
d) Anticouagulants								
e) Corticoesteroids								
f) Fluids								
g) Blood samplimg								
h) Diuretics								

(unknown=99/99) *(00:00-23:59 hrs)*

F) OTHER COMPLICATIONS

28) Did the woman have any complication during hospital stay? ***(Refer to Q26 in GLOSS form)*** **No** [ ] **Yes** [ ]

If No, go to Q18

28.1) If the woman had any of the following complications, date and time of diagnosis

	d	d	mo	mo	h	h	mi	mi
a) Embolic disease								
b) Obstructed labour / dystocia								
c) Anaesthetic complication								
d) Other surgical complications								

29) Did the woman meet any of the near-miss criteria during hospital stay? **No** [ ] **Yes** [ ]

If No, go to Q19 ***(Refer to Q29 in GLOSS form)***

If Yes, specify below:

a) Cardiovascular dysfunction **No** [ ] **Yes** [ ]

a.1) If Yes, date and time first criteria was met

d	d	m	m	y	y		h	h	m	m

(unknown=99/99/99) *(00:00-23:59 hrs)*

b) Respiratory dysfunction **No** [ ] **Yes** [ ]

b.1) If Yes, date and time first criteria was met

d	d	m	m	y	y		h	h	m	m

(unknown=99/99/99) *(00:00-23:59 hrs)*

c) Renal dysfunction **No** [ ] **Yes** [ ]

c.1) If Yes, date and time first criteria was met

d	d	m	m	y	y		h	h	m	m

(unknown=99/99/99) *(00:00-23:59 hrs)*

d) Coagulation dysfunction **No** [ ] **Yes** [ ]

d.1) If Yes, date and time first criteria was met

d	d	m	m	y	y		h	h	m	m

(unknown=99/99/99) *(00:00-23:59 hrs)*

e) Hepatic dysfunction **No** [ ] **Yes** [ ]

e.1) If Yes, date and time first criteria was met

d	d	m	m	y	y		h	h	m	m

(unknown=99/99/99) *(00:00-23:59 hrs)*

d) Neurologic dysfunction **No** [ ] **Yes** [ ]

d.1) If Yes, date and time first criteria was met

d	d	m	m	y	y		h	h	m	m

(unknown=99/99/99) *(00:00-23:59 hrs)*

e) Uterine dysfunction **No** [ ] **Yes** [ ]

e.1) If Yes, date and time first criteria was met

d	d	m	m	y	y		h	h	m	m

(unknown=99/99/99) *(00:00-23:59 hrs)*

G) ADMISSION TO ICU OR HIGH DEPENDENCY CARE

30) Was the woman admitted to ICU or high dependency care? No ☐ Yes ☐

(Refer to Q30 in GLOSS form - October 2017)

If No, go to question Q41

31) If Yes, date and time of admission

d	d	m	m	y	y

(unknown=99/99/99)

h	h	m	m

(00:00-23:59 hrs)

32) What was the main diagnosis at admission to ICU/ high dependency unit?

	No	Yes
a) Haemorrhage	☐	☐
b) Pregnancy related hypertension	☐	☐
c) Infection	☐	☐
d) Other	☐	☐

d.1 If other, specify: *(refer to list)* ____________________

After suspicion/diagnosis of infection

(date of Q15 in GLOSS form - October 2017)

33) Did the woman have hypotension

(SBP<90mmHg/ SBP decrease of ≥40mmHg/ MAP<65mmHG)

Unknown ☐ No ☐ Yes ☐

34) Did the woman have a serum lactate ≥ 4 mmol/L?

Unknown ☐ No ☐ Yes ☐

34.1) If yes to Q33 or Q34, did the woman received crystalloids≥30ml/Kg?

(Ringer, exclud. glucose)

	Unknown	No	Yes
a) No fluids delivered	☐	☐	☐
b) Yes, but not within the first 24h	☐	☐	☐
c) Yes, within the first 24h	☐	☐	☐

34.2) If crytalloids received,date and time of first administration

d	d	m	m	y	y

(unknown=99/99/99)

h	h	m	m

(00:00-23:59 hrs)

35) Did MAP rise to and remain ≥65 after initial fluid resuscitation? No ☐ Yes ☐

36) Did MAP remain≥65 without the use of vasoppressors? No ☐ Yes ☐

37) Central line inserted No ☐ Yes ☐

37.1) If yes date and time

d	d	m	m	y	y

(unknown=99/99/99)

h	h	m	m

(00:00-23:59 hrs)

37.2) If yes, central venous pressure measured No ☐ Yes ☐

37.3) If yes, central venous pressure ≥8 mm Hg No ☐ Yes ☐

37.4) If yes date and time

d	d	m	m	y	y

(unknown=99/99/99)

h	h	m	m

(00:00-23:59 hrs)

38) Was passive leg raising test performed before initiation of fluid therapy ? No ☐ Yes ☐

39) Did the woman receive the following

	No	Yes
a) Vasopressors (applied for hypotension not responding to initial fluid resuscitation)	☐	☐
b) Hydrocortisone (applied for hypotension not responding to fluid resuscitation and vasopressor)	☐	☐
c) Insulin (applied when 2 consecutive blood glucose levels were >180)	☐	☐
d) Intravenous immunoglobulins	☐	☐
e) Antithrombin	☐	☐
f) Heparin	☐	☐
g) Erythropoietin	☐	☐
h) Stress ulcer prophylaxis (proton pump inhibitors, H2RAs)	☐	☐
i) Platelet transfusion received (with counts <10,000 mm3)	☐	☐
j) Thromboprophylaxis	☐	☐
k) Red blood cell transfusion received (with haemoglobin concentration <7.0 g/dL)	☐	☐
l) Fresh frozen plasma (received for correction of clotting abnormalities)	☐	☐
m) Any enteral feeding restriction	☐	☐
n) Parenteral nutrition	☐	☐

If woman admitted to ICU or high dependency care, complete daily record with data on day of admission, -1 and +1

GLOBAL MATERNAL SEPSIS STUDY - DEATHS AND NEARMISS

World Health Organization

INDIVIDUAL FORM - ALL WOMEN ADMITTED TO ICU OR HIGH DEPENDECY CARE

Country code | | | | Hospital ID | | | Participant number | | |

Daily Record - Section H (March 2019 - 6/8 page)

Global Maternal Sepsis Study

			**Day -1** (immediate previous calendar day)	**Day 0** (day of admission to ICU) *Same as Q35*	**Day +1** (immediate subsequent calendar day)
		**Date**	d d m m	d d m m	d d m m
40. Record the most extreme value or corresponding answer at each calendar day			Enter values below. Enter a dash (–) if not measured or unknown. If only measured once during a day record the unique value as lowest value		
**Clinical signs**	40.1.a	(YES / NO) **Altered mental status**			
	40.1.b	**Lowest Glasgow Coma Score**			
	40.2.a	**Highest temperature - °C**			
	40.2.b	**Lowest temperature - °C**			
	40.3.a	(breaths per minute) **Highest respiratory rate - bpm**			
	40.3.b	(breaths per minute) **Lowest respiratory rate - bpm**			
	40.4.a	(beats per minute) **Highest heart rate - bpm**			
	40.4.b	(beats per minute) **Lowest heart rate - bpm**			
	40.5.a	**Highest SBP - mmHg**			
	40.5.b	**Lowest SBP - mmHg**			
	40.6	**Lowest DBP - mmHg**			
	40.7	(YES / NO) **Urine passing in 24h**			
	40.8	(ml/24h) **Urine output**			
	40.9	(lowest O2 saturation) **Pulse-oxymetry (%)**			
	40.1	(YES / NO) **O2 supplementation at time of pulse-oxymetry**			
**Hemogram**	40.11	(lowest value) g/dL **Haemoglobin**			
	40.12	(lowest value) **Hematocrit - %**			
	40.13	**WBC count - mm3**			
	40.14	(highest value) **% of immature neutrophils (bands)**			
	40.15	(lowest value) **Platelet count - x10^3 ml**			
**Gasometry** (enter lowest value)	40.16	**pH**			
	40.17	**Lowest O2 saturation (%)**			
	40.18	**Oxygen partial pressure (PaO2)**			
	40.19	**PaCO2 - mmHg**			
	40.20	**Bicarbonate HCO3 - mEq/L**			
	40.21	**Base excess**			
	40.22	**FiO2 at the time of gasometry**			
**Other laboratory exams** (enter highest value)	40.23	(highest value) **Bilirubin - mg/dL**			
	40.24	**Creatinine - mg/dL**			
	40.25	**Urea - mg/dL**			
	40.26	**Lactate - mg/dL**			
	40.27	**Glucose - mg/dL**			
	40.28	**PT (prothrombin time) - sec**			
	40.29	**aPTT (activated partial thromboplastin time) - sec**			
	40.30	**CRP - mg/L**			
	40.31	**Procalcitonin test - µg/L**			
	40.32	**Erythrocyte sedimentation rate**			
**Other clinical signs** (YES / NO)	40.33	**Decreased capillarity refill or mottling**			
	40.34	**Ileus (absent of bowel sounds)**			
	40.35	**Jaundice**			
	40	**Fetal heart rate > 160 - bpm**			
**Management** (enter highest dosage/number)	40.37	(0=No, 1=<5 µg; 2=5-10 µg; 3=>10 µg/kg/min; 4=unknown dose) **Dopamine**			
	40.38	(0=No; 1=<0.1µg; 2=>0.1 µg/kg/min; 4=unknown dose) **Epinephrine or Norepinephrine**			
	40.39	(YES / NO) **Dobutamine**			
	40.40	(YES / NO) **Vasopressin**			
	40.41	(0=NO; 1=nasal catheter; 2=facial mask; 3=non-invasive ventilation; 4=intubation) **Supplemental Oxygen**			
	40.42	**Received fluids in the event of SBP < 100mmHg** * excludes Glucose 5% (0=No, 1=<1L; 2=1-2L; 3=>2L; 4=unknown dose)			

I) PREGNANCY LOSS (ABORTION, ECTOPIC PREGNANCY)

41) Did the woman have an abortion or ectopic pregnancy? No ☐ Yes ☐

(Refer to Q31 in GLOSS form - October 2017)

If No, go to Q44

41.1) Date symptoms started

d	d	m	m	y	y

(unknown=99/99/99)

42) Expulsion of products of conception ocurred before arrival in any health facility? Unknown ☐ No ☐ Yes ☐

43) Did medical record indicate if this was an induced abortion? Unknown ☐ No ☐ Yes ☐

J) IF THE WOMAN HAD A STILLBIRTH OR LIVEBIRTH

44) Did the woman had a caesarean section? *If No, go to Q48* No ☐ Yes ☐

44.1) If yes, was caesarean section

	No	Yes
a) Emergency	☐	☐
b) Planned	☐	☐

44.2) If yes, please provide indication: *(refer to list)*

____

____

45) Who performed the caesarean section?

- a) Obstetrics specialist ☐
- b) Obstetrician in training (resident) ☐
- c) Physician who can perform caesarean section ☐
- d) Other ☐
- d.1) If other, specify:

____

____

46) What technique was used for the caesarean delivery?

- a) Midline infraumbilical incision ☐
- b) Vertical incision ☐
- c) Transverse incision ☐
  (e.g Pfannenstiel, Mayland, Joel Cohen)

47) Date and time woman was discharged from labour ward or recovery room after CS?

d	d	m	m	y	y		h	h	m	m

(unknown=99/99/99) *(00:00-23:59 hrs)*

K) IF WOMAN ENTERED THE STUDY POSTPARTUM

48) Did she give birth in this facility? Unknown ☐ No ☐ Yes ☐

If No, go to Q52

48.1) If yes, date and time of discharge after childbirth

d	d	m	m	y	y		h	h	m	m

(unknown=99/99/99) *(00:00-23:59 hrs)*

48.2) If yes, diagnosis at discharge

____

L) DEATH (leave blank if nearmiss)

49) Date and time of death

(Refer to Q33 in GLOSS form - October 2017)

d	d	m	m	y	y		h	h	m	m

(00:00-23:59 hrs)

50) Cause of death as in death certificate

a) Immediate cause ________________
Due to (or as a consequence of):

b) Underlying cause ________________
Due to (or as a consequence of):

c) Last cause ________________
Due to (or as a consequence of):

____

51) Was an autopsy performed? Unknown ☐ No ☐ Yes ☐

51.1) If yes, main results of autopsy

____

____

52) Was the death classified using ICD system?

- a) No ☐
- b) Yes, using ICD 10 ☐
- c) Yes, using ICD-MM ☐

53) Please provide ICD codes as in facility registers

- a) Primary cause __________
- b) Final cause of death __________
- c) Contributory (Antecedent) cause 1 __________
- d) Contributory (Antecedent) cause 2 __________
- e) Contributory (Antecedent) cause 3 __________

World Health Organization

Global Maternal Sepsis Study

COMMENTS ______________________

____

____

____

____

Q16. Antimicrobials received ***(Refer to Q24 in GLOSS form)***

Amikacin	Erythromycin
Amoxicillin	Gentamycin
Ampicillin	Linezolid
Azitromicin	Methicillin/ Oxacillin
Carbapenems	Metronidazole
Cephalosporin (1st, 2nd gen)	Penicilin G
Cephalosporin (3rd, 4th gen)	Norfloxacin
Ciprofloxacin	Nitrofurantoin
Clarithromycin	Piperacillin
Clindamycin	Piperacilina/tazobactam
Co-amoxiclav	Polymyxin B/Colistin
Doxycycline	Vancomycin

Q37. Diagnosis at admission to ICU

Cardiac disease	Diabetic ketoacidosis
Haemorraghe	Liver disease
Pregnancy related hypertension	Renal disease
Eclampsia/Hellp	Embolism
Acute respiratory failure	Cerebrovascular accident
Pulminary oedema	Trauma
Infection	Anesthesia complications

Q47.2. Indications of caesarean section

Suspected fetal growth impairment
Fetal distress
Fetal death
Prelabour rupture of membranes
Chorioamnionitis
Pre-eclampsia/eclampsia
Gestational age 41 completed or more
3rd trimester vaginal bleeding
Cephalopelvic disproportion
Failure to progress/labour dystocia
Multiple pregannacy
Suspected/inmminent uterine rupture
Postmortem CS
Previous CS
Breech or other malpresentation
Failed induction
Maternal request
HIV positive
Previously repaired vesico-vaginal/recto-vaginal fistula
Previous uterine surgery

Instructions

a) This form is composed of sections. The target population of each section is specified in the section title
b) Sections or questions may be skipped considering the individual participant. Do not leave blank fields except if indicated
c) Mark the most appropriate answer with an X
d) Please use code list provided to complete free text cells in questions Q16, Q37, Q47.2
If response not in the list, please add correct response
e) Information is to be obtained from hospital records or by questioning the attending staff for any missing information

Data Collector's name and date of closure of the individual forms:

____

d	d	m	m	y	y

Data Entry Operator's name and date:

____

d	d	m	m	y	y

Figure S2: Maternal death and near-miss review clinical summary form template

**Admission to hospital (final point of care)**	**Main reason for admission:**

**Timing of chain of events**		**Patient code:**
	**Date and time of admission at first point of care**:	
	**Date and time of admission to GLOSS facility/study**:	
	**Date and time of suspicion or diagnosis of infection:**	
	**Date and time of first antibiotics:**	
	**Date and time of abortion or delivery:**	
	**Date and time of ICU admission:**	
	**Date and time of theatre admission:**	
	**Date and time of death:**	
	**Time elapsed between complication* and death:**	

*Complication is defined as the first near-miss criteria such as respiratory-, cardiovascular-, renal, coagulation, neurologic, hepatic and uterine dysfunction.

**Maternal characteristics and obstetrical history**	**Patient Age:**	**Marital status:**	
	**Gravida:**	**Para:**	**Live children:**
	**Number of previous caesarean sections:**	**Date of last CS:**	
	**Number of ANC visits in this pregnancy:**		
	**Risk factor(s)/complications detected during this pregnancy/labour/postpartum period:**		
**Case summary presented to the committee**			

**Admission details to Facility 1 (first point of care)**		**Date:**
	**Reason for coming to hospital:**	
	**Pregnant on admission?**	**Duration of amenorrhea:**
	**Alive baby?**	
	**Complications occurred?**	
	**Referred from another institution?**	**Type of institution?**
	**History of the referral/process of reaching the institution:**	

**Admission details GLOSS Facility**		**Date:**
**Admission details GLOSS Facility**	**Main reason for admission:**	
	**Diagnosis made at admission:**	

	**If delivered/aborted before admission:**	**Date:**
	**Place of birth/abortion?**	**Assisted by:**
	**Alive baby?**	
	**Complications occurred?**	
	**Referred from another institution?**	**Type of institution?**
	**Initial clinical assessment/Ultrasound/laboratory findings at admission:**	

**Summary of the case evolution at GLOSS facility**	**Summary of the case evolution if complication(s) occurred after admission:**<br><br>**Complications:**<br>**Clinical assessment/Ultrasound/laboratory findings:**<br><br>**Hemogram:**<br><br>**Other labs:**<br><br>**Diagnosis:**<br><br>**Complementary tests and laboratory results after treatment:**<br><br>**Summary of case evolution and monitoring put in place (t°, BP, Pulse, and Bleeding):**<br><br>**Date of death:**<br>**Time elapsed between complication and death:**<br><br>**Cause of death notified in records:**<br><br>**Pregnancy outcome (Live birth, SB, Early death, Miscarriage):**

**Other information available**	**From family, health centers, community, etc.:** None

**Tentative ICD-MM Coding**	
ICD-MM Code	
Title of group	
Direct cause of death	
Antecedent cause of death/due to or as a consequence of	
ICD-MM Coding	

Figure S3: Note taking form for GLOSS external review meeting

**Case ID**					
**What is your main diagnosis for this case?**					
**Case management problems (CMP) related to:**	Severity of CMP				
	**N/A**[1]	**Minor**[2]	**Intermediate**[3]	**Major**[4]	**Comment**
Before referral or admission to hospital					
woman's ANC unsatisfactory (onset and quantity)					
received unskilled antenatal care (care from traditional birth attendants)					
high risk status (with 1 or more known pre-existing condition).<br>*For example, gestational or chronic hypertension or diabetes, anemia, HIV and other chronic diseases*					
Referral to hospital					
referral delayed (>1 referral or >3o mins interval)					
transport not "medicalized" (not an ambulance)					
Establishing a diagnosis/monitoring					
initial clinical examination unsatisfactory					
initial laboratory assessment unsatisfactory					
main diagnosis wrong					
main diagnosis delayed					
main diagnosis incomplete					
**Diagnostic discrepancies classification (see below)**					
subsequent clinical examination unsatisfactory					
subsequent laboratory assessment unsatisfactory					
secondary diagnosis wrong					

[1] N/A: Not applicable as it did not occur in this case
[2] Minor: Not directly relevant for maternal survival or avoidance of long-term maternal morbidity
[3] Intermediate: Some relevance for maternal survival or avoidance of long-term maternal morbidity
[4] Major: Immediate danger for maternal survival or avoidance of long-term maternal morbidity

secondary diagnosis incomplete					
**Diagnostic discrepancies class (see below for classification)**					
monitoring insufficient					
others_________________________________					
Treatment					
infection					
culture missing					
culture incomplete					
antibiotics delayed					
antibiotics missing					
antibiotics incomplete					
antibiotics wrong/not ideal					
antibiotics overuse/ regimen too broad					
antibiotics unnecessary					
antibiotics resistance detected in culture					
post-culture antibiotics adjustment unsatisfactory					
prophylactic antibiotics for medical procedure missing					
others_____________________________					
general					
drug delayed					
drug missing					
drug overdosed					
drug underdosed					
drug unnecessary					
drug wrong/not ideal					
intravenous replacement fluids delayed					
intravenous replacement fluids missing					
intravenous replacement fluids wrong/not ideal					

intravenous replacement fluids insufficient					
intravenous replacement fluids unnecessary					
blood transfusion delayed					
blood transfusion missing					
blood product transfused wrong/not ideal					
blood transfused insufficient					
blood transfusion unnecessary					
procedure delayed					
procedure missing					
procedure carried out unsatisfactorily					
procedure wrong					
procedure unnecessary					
ICU/HDU transfer delayed					
ICU/HDU transfer missing					
other ___________________________________					
**Managing team or other**					
managing team incomplete					
other____________________________________					
**Additional documents**					
autopsies done for deaths					
death certificate issued					
internal death review /confidential enquiry conducted					
other____________________________________					

Case summary:

Summary of case discussion:

Recommendations:

Diagnostic discrepancies are classified as major or minor*.*
Major discrepancies are classified as class I or class II*.*

- *Class I refers to discrepancies in which the knowledge of the correct diagnosis before death would have led to changes in clinical management that could have prolonged survival or cured the patient (e.g., pyogenic meningitis treated as eclampsia).*
- *In class II errors, patient survival would have not been modified (e.g., fulminant hepatitis treated as sepsis).*

Minor discrepancies involved minor diagnoses and are classified as

- *Class III (non-diagnosed diseases with symptoms that should have been treated—e.g., mild aspiration pneumonia in a patient with eclampsia) and,*
- *Class IV (non-diagnosed diseases with possible epidemiological or genetic importance—e.g., schistosomal infections).*
- *Correctly diagnosed patients are classified as class V.*
- *Class VI comprised non-classifiable cases (autopsy unsatisfactory or with no clear diagnosis).*

Glossary

ANC – Antenatal Care

CMP- Case management problems

ICU- Intensive Care Unit

HDU- High Dependency Unit

Table S1: | Reported performance of maternal death review process in 20 of 25 participating facilities prior to the GLOSS study

	Yes	No	Unsure	Missing
	n(%)	n(%)	n(%)	n(%)
Formal System exists to review maternal deaths				
National level	12(60.0)	5(25.0)	1(5.0)	2(10.0)
District level	12(60.0)	4(20.0)	1(5.0)	3(15.0)
Facility level	14(70.0)	5(25.0)	0	1(5.0)
Formal system exists to review				
Maternal near-miss	10(50.0)	6(30.0)	1(5.0)	3(15.0)
Stillbirths	10(50.0)	4(20.0)	2(10.0)	4(20.0)
Neonatal deaths	16(80.0)	0	2(10.0)	2(10.0)
Presence of MDR guidelines	17(85.0)	-	1(5.0)	2(10.0)
All maternal deaths are reviewed	12(66.7)	6(33.3)	n/a	n/a
Who attends MDR meetings by function (N=18)				
Steering committee only	7(38.9)	11(61.1)	n/a	n/a
Ad-hoc committee	6(33.3)	12(66.7)	n/a	n/a
Staff involved in case management	13(72.2)	5(27.7)	n/a	n/a
Community representatives	3(16.7)	15(83.3)	n/a	n/a
Open to all staff	8(44.4)	10(55.6)	n/a	n/a
Others	7(38.9)	11(61.1)	n/a	n/a
Source of information used for MDR				
Case notes (includes partograph and nursing charts)	18(90.0)	2(10.0)	n/a	3(15.0)
Facility registers	16(80.0)	1(5.0)	n/a	3(15.0)
Death registers	9(45.0)	8(40.0)	n/a	3(15.0)
Death certificates	9(45.0)	8(40.0)	n/a	3(15.0)
Antenatal/MCH cards	13(65.0)	5(25.0)	n/a	2(10.0)
Staff interviews	11(55.0)	6(30.0)	n/a	3(15.0)
Family/Community Interviews	11(55.0)	7(35.0)	n/a	2(10.0)

Community Records	3(15.0)	15(75.0)	n/a	2(10.0)
None	0	17(85.0)	n/a	3(15.0)
Others	2(10.0)	15(75.0)	n/a	3(15.0)
Type of written documentation produced during maternal death review meetings				
Standardized forms	12(60.0)	5(25.0)	n/a	3(15.0)
Written minutes for each case	13(65.0)	4(20.0)	n/a	3(15.0)
Summaries with recommendations and action points	15(75.0)	2(10.0)	n/a	3(15.0)
Standard doc for presenting to mgt	8(40.0)	9(45.0)	n/a	3(15.0)
Annual report with findings from the previous year	5(25.0)	12(60.0)	n/a	3(15.0)
Findings dissemination channels				
Displayed (for example on wall charts or posters)	6(30.0)	11(55.0)	n/a	3(15.0)
Staff Meeting	10(50.0)	7(35.0)	n/a	3(15.0)
Factsheets	4(20.0)	13(65.0)	n/a	3(15.0)
Newsletters/ Bulletins	1(5.0)	16(80.0)	n/a	3(15.0)
Electronic messages	2(10.0)	15(75.0)	n/a	3(15.0)
Press release or media	1(5.0)	16(80.0)	n/a	3(15.0)
Scientific articles	1(5.0)	16(80.0)	n/a	3(15.0)
Website	1(5.0)	16(80.0)	n/a	3(15.0)
Official hospital channels	4(20.0)	13(65.0)	n/a	3(15.0)
Meetings with community leaders or members	2(10.0)	15(75.0)	n/a	3(15.0)
Others	5(25.0)	12(60.0)	n/a	3(15.0)
Individuals assigned to follow up on specific recommendations	15(75)	2(10.0)	n/a	2(10.0)
Implementation tracked by a written documentation system	9(45.0)	9(45.0)	n/a	2(10.0)
Review committee is linked to facility quality Improvement activities	12(60.0)	4(20.0)	2(10.0)	2(10.0)

Table S2 | Distribution of modifiable factors by case

Case No	Prior to arrival at the GLOSS facility	Clinical & laboratory examination	Diagnosis	Management	Managing team	Other factors	Total modifiable factors / case N=151
**Maternal deaths**							
**MD 1**	0	2	2	2	0	0	**6**
**MD 2**	3	0	1	2	0	0	**6**
**MD 3**	1	4	1	4	1	0	**11**
**MD 4**	2	1	1	3	0	0	**7**
**MD 5**	0	2	0	2	0	1	**5**
**MD 6**	1	2	1	2	0	0	**6**
**MD 7**	1	2	1	5	0	0	**9**
**MD 8**	0	2	1	2	1	0	**6**
**MD 9**	1	0	1	1	0	0	**3**
**MD 10**	1	2	1	2	1	0	**7**
**MD 11**	3	3	2	3	1	0	**12**
**MD 12**[1]	-	-	-	-	-	-	-
**MD 13**	0	0	1	5	0	1	**7**
**Maternal near-miss**							
**NM1**	0	0	0	1	0	0	**1**
**NM 2**	1	1	0	1	0	0	**3**
**NM 3**	0	0	2	3	1	0	**6**
**NM 4**	0	1	1	1	0	0	**3**
**NM 5**	1	2	1	2	0	0	**6**
**NM 6**	2	3	1	1	0	0	**7**
**NM 7**	0	0	1	1	0	0	**2**
**NM 8**	2	0	2	1	0	0	**5**
**NM 9**	0	0	0	0	0	0	**0**
**NM 10**	0	0	0	1	0	0	**1**
**NM 11**	0	1	0	1	0	0	**2**
**NM 12**	1	0	0	0	0	0	**1**
**NM 13**	0	1	0	1	0	0	**2**
**NM 14**	2	1	0	4	0	0	**7**
**NM 15**	1	2	1	2	0	1	**7**
**NM 16**	0	1	1	0	1	0	**3**
**NM 17**	0	3	0	2	0	0	**5**
**NM 18**	0	0	1	0	0	0	**1**
**NM 19**	2	1	1	0	0	0	**4**

MD = Maternal death; NM = Near-miss
GLOSS=Global Sepsis Study

[1] Death occurred within the first hour of presentation before the managing team commenced clinical management

Table S3 | Summary of the causes of maternal deaths

Case No	Cause of death from facility records	Direct cause of death assigned in the review process	Antecedent cause assigned in the review process	ICD MM coding assigned in the review process
MD 1	Not assigned	Obstetric death of unspecified cause	Other maternal infectious and parasitic diseases complicating pregnancy, childbirth and the puerperium (viral hepatitis)	Group 8/ O95/O98.4
MD 2	Endometritis post-abortion complicated by septic shock	Shock following abortion (circulatory collapse)<br>Septic shock following abortion	Genital tract and pelvic infection following abortion and ectopic and molar pregnancy (Endometritis)	Group 1/ O08.3/O08.0 (R57.2)/O08.1
MD 3	Bowel obstruction post cesarean complicated with shock	Puerperal sepsis (septic shock)		Group 4/O85
MD 4	Cardiorespiratory arrest; Multiorgan failure<br>Refractory septic shock	Septic shock (Circulatory collapse), multiple organ failure	Puerperal sepsis (Uterine infection)<br>Postpartum coagulation defects<br>Postpartum - Hepatorenal syndrome following labour and delivery	Group 2/ O85/O72.3 /O90.4
MD 5[6]	Acute Pulmonary Embolism	Obstetric pulmonary embolism	Puerperal sepsis (peritonitis)	Group 5/ O88.2/O85/ O98.7
MD 6	Infection	Incomplete spontaneous abortion complicated by genital tract and pelvic infection	Diseases of the respiratory system (Respiratory tract Infection)<br>Possible pre-eclampsia	Group 1/ O08.0/O99.5/ O14
MD 7	Disseminated Intravascular Coagulation (DIC)<br>Septic abortion<br>Severe anemia	Hemorrhagic shock (Circulatory collapse)	Delayed or excessive hemorrhage following abortion and ectopic and molar pregnancy<br>Genital tract and pelvic infection following abortion and ectopic and molar pregnancy	Group 1/ O08.1/O08.0/ O02.1

[6] Contributory cause of death assigned: HIV complicating pregnancy and childbirth

Case No	Cause of death from facility records	Direct cause of death assigned in the review process	Antecedent cause assigned in the review process	ICD MM coding assigned in the review process
			Missed abortion. Early fetal death with retention of dead fetus	
MD 8	Infection	Septic shock	Infections of kidney in pregnancy (pyelonephritis)	Group 4/ R57.2/O23.0
MD 9	Immediate cause: complications of spinal anesthesia Underlying cause: congenital heart disease	Complications of anesthesia during labor and delivery	Preexisting disease of the respiratory system (Congenital heart disease, asthma)	Group 6/O74
MD 10	Infection	Septic shock	Sepsis Cardiomyopathy	Group 4/ O85/I42
MD 11	Infection	Septic shock Delayed or excessive hemorrhage following abortion	Failed attempted abortion	Group 1/ R57.2/O08.1/O07
MD 12	Not assigned	Septic shock	Puerperal sepsis (Uterine infection)	Group 4/O85
MD 13	Immediate cause: Post abortion sepsis Underlying cause: Severe pneumonia Last cause: Cushing syndrome	Septic shock following abortion	Incomplete abortion complicated by genital tract and pelvic infection	Group 1/ R57.2/O08.0
